# Active proportional electromyogram controlled functional electrical stimulation system

**DOI:** 10.1038/s41598-020-77664-0

**Published:** 2020-12-04

**Authors:** Bethel A. C. Osuagwu, Emily Whicher, Rebecca Shirley

**Affiliations:** 1 National Spinal Injuries Centre, Stoke Mandeville Hospital, Mandeville Road, Aylesbury, HP21 8AL UK; 2 Buckinghamshire Healthcare Plastics, Stoke Mandeville Hospital, Mandeville Road, Aylesbury, HP21 8AL UK

**Keywords:** Neuromuscular disease, Biomedical engineering

## Abstract

Neurophysiological theories and past studies suggest that intention driven functional electrical stimulation (FES) could be effective in motor neurorehabilitation. Proportional control of FES using voluntary EMG may be used for this purpose. Electrical artefact contamination of voluntary electromyogram (EMG) during FES application makes the technique difficult to implement. Previous attempts to date either poorly extract the voluntary EMG from the artefacts, require a special hardware or are unsuitable for online application. Here we show an implementation of an entirely software-based solution that resolves the current problems in real-time using an adaptive filtering technique with an optional comb filter to extract voluntary EMG from muscles under FES. We demonstrated that unlike the classic comb filter approach, the signal extracted with the present technique was coherent with its noise-free version. Active FES, the resulting EMG-FES system was validated in a typical use case among fifteen patients with tetraplegia. Results showed that FES intensity modulated by the Active FES system was proportional to intentional movement. The Active FES system may inspire further research in neurorehabilitation and assistive technology.

## Introduction

Functional electrical stimulation (FES) is a technique used for movement rehabilitation^1,2^. It can be used to activate muscles allowing paralysed people with conditions such as spinal cord injury (SCI) and stroke to perform functional tasks in a manner that may lead to motor recovery^2^. Conventionally FES is applied passively such that a user has no control over an ongoing activation of a muscle. Instead a preprogramed pattern is used which allows the user to only switch on/off the FES device. Current literature suggests that this application technique shows inconclusive results in movement rehabilitation^3^. The reason for this could be due to the passive nature of the technique as neurophysiological theories and evidence suggest that active engagement is integral to optimal effect of FES^4–7^.

**Figure 1:**
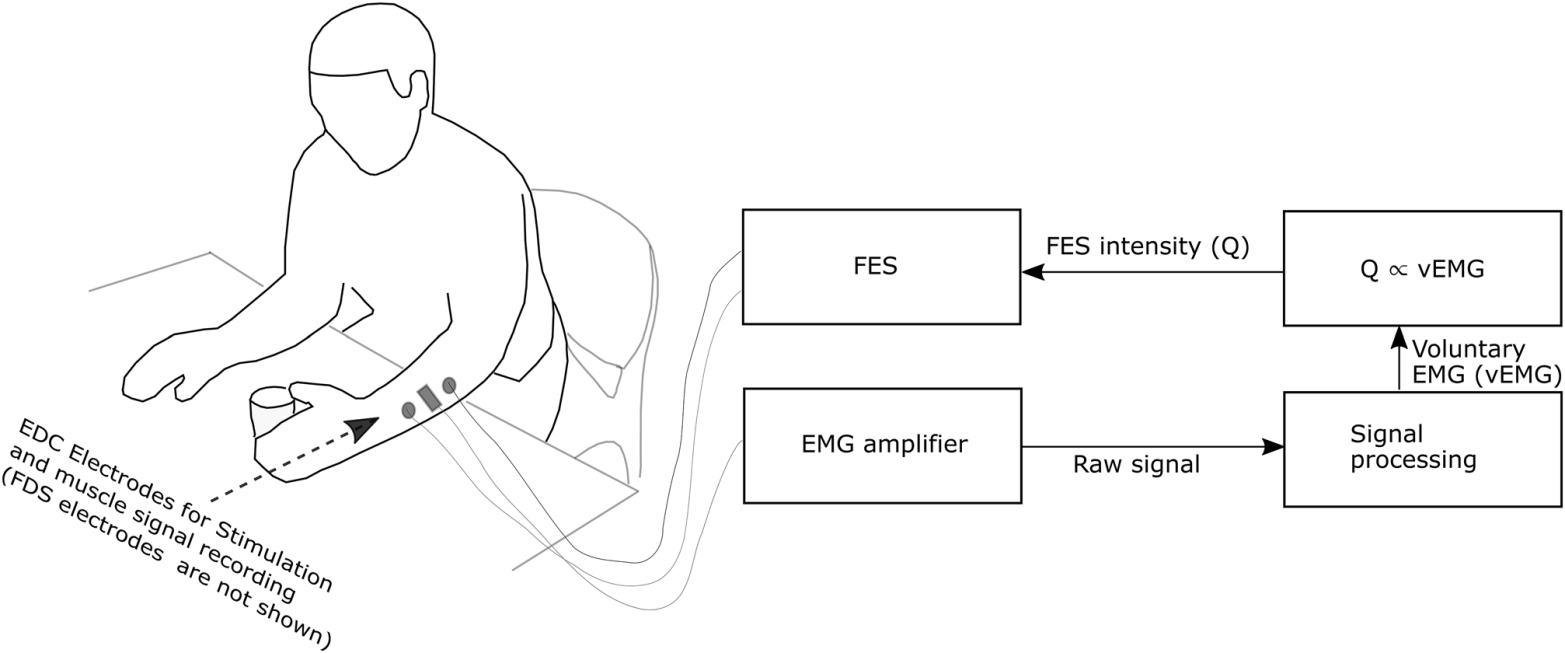
The schematic diagram of a proportional EMG-FES system. EDC, Extensor digitorum communis muscle of the hand; FDS, Flexor digitorum superficialis muscle of the hand; FES, Functional electrical stimulation; EMG, Electromyogram.

In order to engage a user during FES a method of triggering the stimulation following the detection of a set level of electromyogram (EMG) has previously been studied^8–12^. Electromyogram provides information on neural activities of a muscle and may be used to detect movement intention^13^. With this method stimulation at a set level is delivered when EMG magnitude meets a set threshold. This basic implementation of the active engagement which only necessitates a user’s action at the start of the stimulation by triggering FES after detecting muscle activities has not conclusively demonstrated an advantage over the passive method^8–12^. However functional electrical therapy (FET)^1,2^, FES therapy^14^ and brain computer interface controlled-FES (BCI-FES)^15^ protocols which encourage a user to continuously attempt movement along with an ongoing electrical stimulation have led to improvement in motor function. This suggests that explicit necessitation of a user’s engagement during FES may be better implemented using a proportional^16^ instead of triggered method of FES application. With the proportional method, shown schematically in Fig. 1, voluntary EMG (vEMG) is continuously extracted from a stimulated muscles and used to modulate the intensity $Q$ of the ongoing stimulation ($Q\propto \left|EMG\right|$). The occurrence of stimulation related artefacts in the EMG recorded during FES has made this method difficult to implement. A solution for this issue is required in order to implement an effective proportional method.

The stimulation related artefacts affect EMG recorded from the proximity of an ongoing electrical stimulation, and are namely (1) transient stimulation^17^, (2) post stimulus voltage-decay^18^ and (3) m-wave^17^. Note that these are regarded as artefacts here since they obscure the vEMG. The transient stimulation artefact is a sharp, large amplitude direct contamination from the electrical stimulation. The delivered charge from the stimulation dissipates slowly affecting a few signal samples and leads to the post-stimulus voltage decay. The m-wave artefact arises from coordinated evoked response of neurones that innervate the stimulated muscles.

Several techniques exist in the literature for extraction of vEMG from a muscle under FES^19–21^. However, only a few techniques have been demonstrated to work online. This is likely due to the difficulty involved in the related real-time signal processing. The simplest techniques that work online use blanking windows, fixed response comb and bandpass/high-pass filters to eliminate artefacts^22–25^. The techniques are often unreliable because the fixed filters are not adequate to deal with the non-stationary artefact encountered in signals recorded during FES. Furthermore the use of a blanking window leads to loss of data within the window. An adaptive method which estimated the stimulation related artefact by filtering the recorded EMG with a recursive filter has been investigated to deal with the non-stationary nature of the artefacts^26^. The output of the filter was subtracted from the original input signal to leave vEMG behind. Although the method enabled adaptation to the changing artefacts, the presented data showed that the artefacts were not entirely eliminated. In 1997 Sennels et al.^17^ dealt with the problem by implementing an adaptive filtering technique to eliminate the non-stationary m-wave artefact. They also used a hardware pre-processor which implements a blanking window to eliminate the transient stimulation artefact. Although the blanking window created dead zones in the signal they found that the technique extracted signal adequate for the control of the FES that induced the artefact.

Following Sennels et al. in 1997, devices by Thorsen and Muraoka were developed between 1999 and 2002^27,28^. These devices are similar to each other except that Muraoka’s uses the same electrode for both stimulation and recording. More recently, in 2011 Shalaby et al.^29^ developed a similar device to that by Muraoka but with improved bandwidth of the extracted voluntary EMG signal. These devices were designed to focus on estimating vEMG adequate for control purposes. They concentrated on the major transient stimulation artefact using a blanking window to eliminate it. The use of a blanking window, the length and periodicity of which are functions of FES pulsewidth and frequency respectively, means that the estimated signal has missing information within the windows. By extending the length of the blanking window, part of the m-wave and post-stimulus decay can be removed but this increases the dead zones with more loss of data. Furthermore, the techniques relying on a blanking window often require a high-pass filter with high frequency cutoff. For example, high-pass cutoff frequencies of 300 Hz, 100 Hz and 170 Hz were used respectively for the devices by Muraoka^28^, Thorson^30^ and Shalaby^29^. Such high frequency cutoffs will likely reduce the energy of the extracted vEMG. Hence, these devices were not able to extract pure vEMG from the contaminated signal without significant loss of data and may not be optimal for studying the target muscle due to unfiltered artefacts.

The active functional electrical stimulation (Active FES) system presented here is an online entirely software-based solution that can both adaptively extract vEMG from muscles under FES and proportionally modulate the intensity of the stimulations using the extracted signal. The System which does not require a blanking window and a high frequency cutoff is controlled through a graphical user interface and incorporates tracing tasks and a visual feedback interface showing detected movement intention and FES intensities on each stimulated muscle. Simulation results show that the signal extracted with the Active FES system is more coherent with the real vEMG than that extracted with the classic comb filter. The coherence result suggests that the signal extracted using the Active FES system is sufficiently similar to the real vEMG. In order to demonstrate the feasibility of the Active FES, we piloted it among fifteen people with chronic and acute tetraplegia who although may have minimal residual EMG, are likely users of such a system for rehabilitation purposes, and demonstrated that the system can proportionally modulate the intensity of FES in accordance with a user’s intentional movement. The patients, despite the minimal residual EMG, found the device usable and rated it 4/5 suggesting that the Active FES may be acceptable for motor rehabilitation among tetraplegic patients. Other potential application areas for the Active FES system include neuroprosthesis, biofeedback and neuroscientific investigation of a muscle under FES.

## Results

### Artefacts, filters and Active FES system

**Figure 2:**
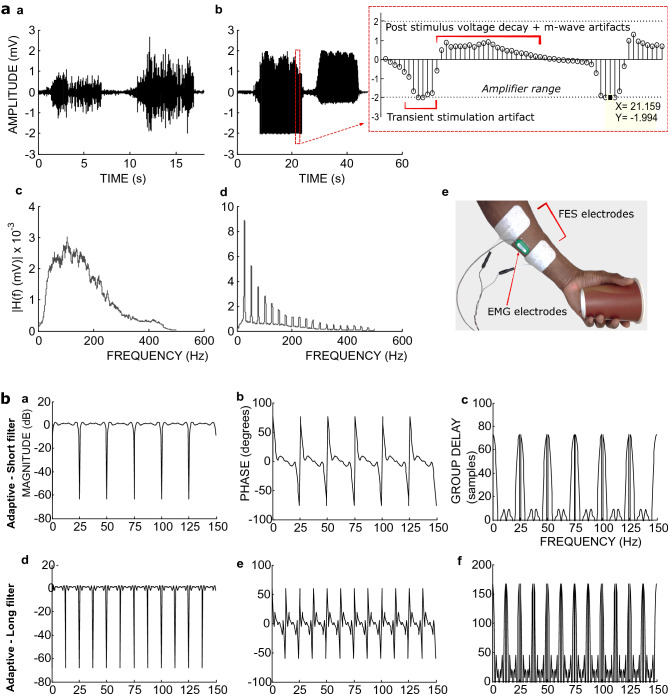
(**a**) Time and corresponding frequency characteristics of the stimulation artefacts, m-wave artefact, and real EMG acquired with FES frequency of 25 Hz and EMG sample rate of 1000 Hz. (**aa**) Time-domain EMG, (**ab**) EMG with real stimulus and m-wave artefacts in time domain. In the inset, the samples in the plot have a period of 0.001 s and the window representing the “Transient stimulation artefact” indicates when stimulation was applied. (**ac**) Frequency spectrum of the EMG in (**aa**). (**ad**) The spectrum of the EMG with real stimulus and m-wave artefacts in (**ab**). (**ae**) Recording setup of the presented signals. (**b**) The Average frequency and phase responses and group delays of the adaptive filters. These show the characteristics when filtering the *simulated* m-wave from the real EMG data (see text). (**ba**) Magnitude response of the short adaptive filter. (**bb**) Phase response of the short adaptive filter. (**bc**) Group delay of the short adaptive filter. (**bd**) Magnitude response of the long adaptive filter. (**be**) Phase response of the long adaptive filter. (**bf**) Group delay of the long adaptive filter. Short filter, length = 41 (order = 40) samples; Long filter, length = 81 (order = 80) samples.

Figure 2aa-ad shows an example EMG and the nature of the effect of stimulation and m-wave artefacts on muscle activities recorded during FES from hand muscles (Fig. 2ae). There is a significant increase in amplitude both in time and in frequency domains of the recorded signal during FES application (Fig. 2ab,ad cf. Figure 2aa,ac). A maximum of four sample values may be clipped due to saturation after a stimulation pulse recorded with our amplifier (Fig. 2ab inset). With EMG sample rate of 1000 Hz and FES frequency of 25 Hz, these accounts for a maximum of 10% of the recorded samples between stimulation pulses (total = 40 samples) but the remaining 90% may be enough information to model the artefacts and the underlying vEMG.

In order to remove the transient electrical stimulation artefact, we designed a ***comb filter*** capable of supressing the 25 Hz frequency and its harmonics. For the post-stimulus voltage decay and m-wave we designed an ***adaptive filter***^17^ to predict an average of these artefacts following every stimulation pulse. To implement the adaptive filter six previous data frames in addition to a current frame were used to construct a vectorised linear least square problem in Simulink (MathWorks, Inc) to estimate the average of the post-stimulus voltage decay and m-wave artefacts for the current data frame. Each data frame contained 40 (for a ***short filter***) or 80 (for a ***long filter***) data samples. The predicted average artefact signal was subtracted from the current frame. We studied the *comb* and *adaptive filter* by examining the corresponding filter coefficients using a real EMG data combined with a simulated stimulation and m-wave artefacts as input.

The results show that the short adaptive filter has a wide passband and removes a narrow band at 25 Hz and its resonance frequencies (Fig. 2ba). This frequency response is similar to that of the long filter barring the bandstop at 12.5 Hz and the resonance frequencies for the long filter (Fig. 2bd). The phase responses (Fig. 2bb,be) are approximately linear across the passband with a discontinuity at the bandstops. Using the group delay (Fig. 2bc,bf) we estimated the likely filter delay as ≈49 ms for the short filter and ≈125 ms for the long filter (see Methods).

**Figure 3:**
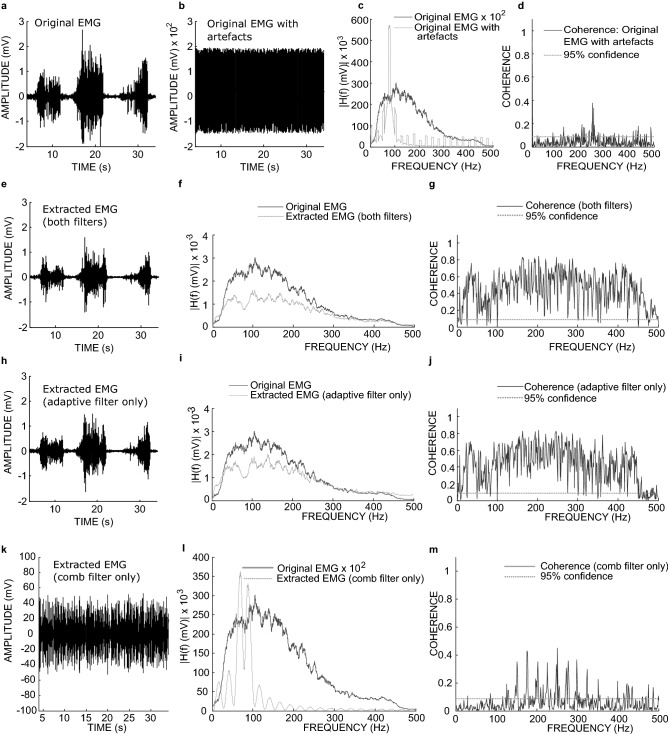
Filtering performance when a real EMG with added simulated stimulation and m-wave artefacts was filtered with the short adaptive, and the comb filter. (**a**) Original EMG. (**b**) The original EMG with simulated stimulation and m-wave artefacts added. (**c**) Spectrum of the original EMG and, the original EMG with stimulus and m-wave artefacts. (**d**) Coherence between the original EMG and, the original EMG with the added artefacts. (**e**) Extracted signal with both the adaptive and comb filter. (**f**) Spectrum of the original and the signal extracted with both the adaptive and comb filter. (**g**) Coherence between the original signal and the signal extracted with both the adaptive and comb filter. (**h**) Extracted signal with only the adaptive filter. (**i**) Spectrum of the original and the signal extracted with only the adaptive filter. (**j**) Coherence between the original and the signal extracted with only the adaptive filter. (**k**) Extracted signal with comb filter. (**l**) Spectrum of the original and the signal extracted with comb filter, (**m**) Coherence between the original signal and the signal extracted with comb filter.

Figure 3 shows the filtering outcome in time and frequency domains when a real EMG (Fig. 3a) mixed with stimulus and m-wave artefacts (Fig. 3b–d) was filtered with the short adaptive filter. The remarkable result is that with (Fig. 3e–g) and without (Fig. 3h–j) the comb filter the adaptive filter was capable of extracting the vEMG from the artefacts. On the other hand, using the comb filter alone, it was not possible to recover the original signal (Fig. 3k–m). Muscles response index (MRI^17^), power reductions and average coherence were used to assess the filtering performance of all filters with summary presented in Table 1. The MRI index measured in dB has a value of zero for a perfect filtering and less than zero for imperfect filtering. When using the short version of the adaptive filter, and the comb filter, the extracted signal was more coherent with the original EMG (0.45, 95% confidence = 0.087) but when used alone the short adaptive filter had the best MRI_y_ of − 0.7 dB (cf. MRI_x_ = − 52.0 dB), and a significant coherence of 0.42 (95% confidence = 0.087). This result suggests that the short adaptive filter can be used alone (without the need for the comb filter) to extract the EMG. When the long adaptive filter was used alone (Supplementary Fig. S1) the extracted signal spectrum seemed to more closely approximate that of the original signal (see Supplementary Fig. S1g) compared with the short filter. Overall the long filter led to a poorer coherence and MRI_y_ values compared with the short filter as shown on Table 1. For the classic comb filter, it had the worst performance with MRI_y_ of − 43.2 dB (cf. MRI_x_ = − 52.0 dB) and a power reduction, relative to the input signal with artefact, of only − 15.0 dB. The output of the comb filter was largely incoherent (0.064, 95% confidence = 0.087) with the original EMG (Fig. 3k–m).

**Table 1:** Filter performance scores.

Filters	MRI_x_ (dB)	MRI_y_ (dB)	PR—extracted vs clean (dB)	PR—extracted vs artefact (dB)	Avg. Coh	Coh. 95% confidence
Both (short adaptive)	− 52.036	− 0.87298	− 5.0815	− 54.726	0.44595	0.086781
Adaptive only (short adaptive)	− 52.036	− 0.70469	− 3.1071	− 52.752	0.41945	0.086781
Both (long adaptive)	− 52.036	− 1.0195	1.5869	− 48.058	0.38264	0.086781
Adaptive only (long adaptive)	− 52.036	− 1.8188	− 1.0888	− 50.733	0.34617	0.086781
Comb	− 52.036	− 43.233	34.633	− 15.012	0.063795	0.086781

*Both filters* refers to the comb and adaptive filter. MRI, muscle response index; MRI_x_, MRI of the input signal; MRI_y_, MRI of the output signal; PR, power reduction; Avg Coh, Average coherence; Coh, coherence.

### Pilot study of the Active FES system in spinal cord injury

**Figure 4:**
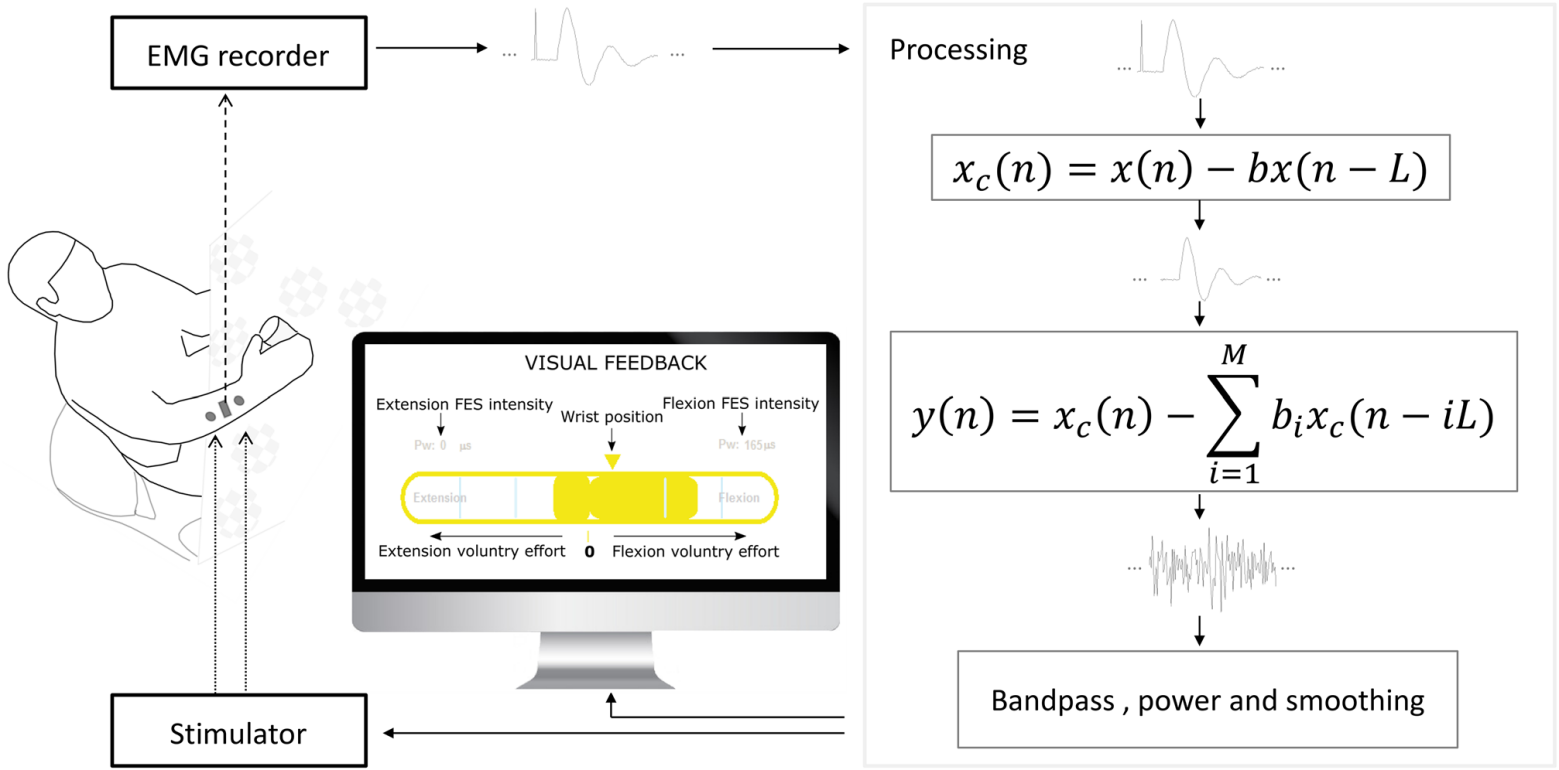
Schematic diagram of the Simulink model showing the Active FES system implementation integrating the filtering with EMG and FES systems. The EMG data from the recorder was entirely passed through the filters in the order shown. The output of the filters which represents voluntary EMG was further processed and used to modulate the intensity of the FES, and as visual feedback. The model also provides a visual feedback through a computer monitor. The flexor electrodes and goniometer which was used to measure the position of the wrist are not shown to avoid clutter.

We implemented the Active FES system shown in Fig. 4, using the filters we have designed to simultaneously record EMG and apply FES on the same muscle. Using the recorded EMG, the system allows a user to voluntarily and proportionally modulate the intensity of FES. The Active FES has two channels each comprising an EMG and an FES channel. It incorporates a visual feedback system which presents wrist position and for each channel the extracted EMG as an indicator of voluntary effort, FES intensity and FES activation threshold (Supplementary Fig. S2).

In order to test the Active FES in a typical use case, we examined its usability among patients with SCI who have limited functional capacity of the hand (Table S2, Table S3, Table S4). Normally the patients should be able to perform a target tracing task using a feedback derived e.g. from their residual vEMG superimposed on the target. Unless the Active FES system can eliminate stimulation related artefacts from the vEMG, the patients’ performance on the task should worsen upon the introduction of FES. Also unless the artefacts are eliminated, FES intensity (pulsewidth) will not correlate with vEMG to give a proportional EMG-FES system. Therefore two hand tracing tasks were used to demonstrate among the patients that the Active FES system can, (1) extract vEMG from stimulated muscles of patients with limited function and (2) use the extracted signal to proportionally control the FES intensity. During the tasks the FES frequency remained 25 Hz with the pulsewidth determined by EMG power in accordance with the Active FES system (see Methods) and current (10–28 mA) set to minimise discomfort.

**Figure 5:**
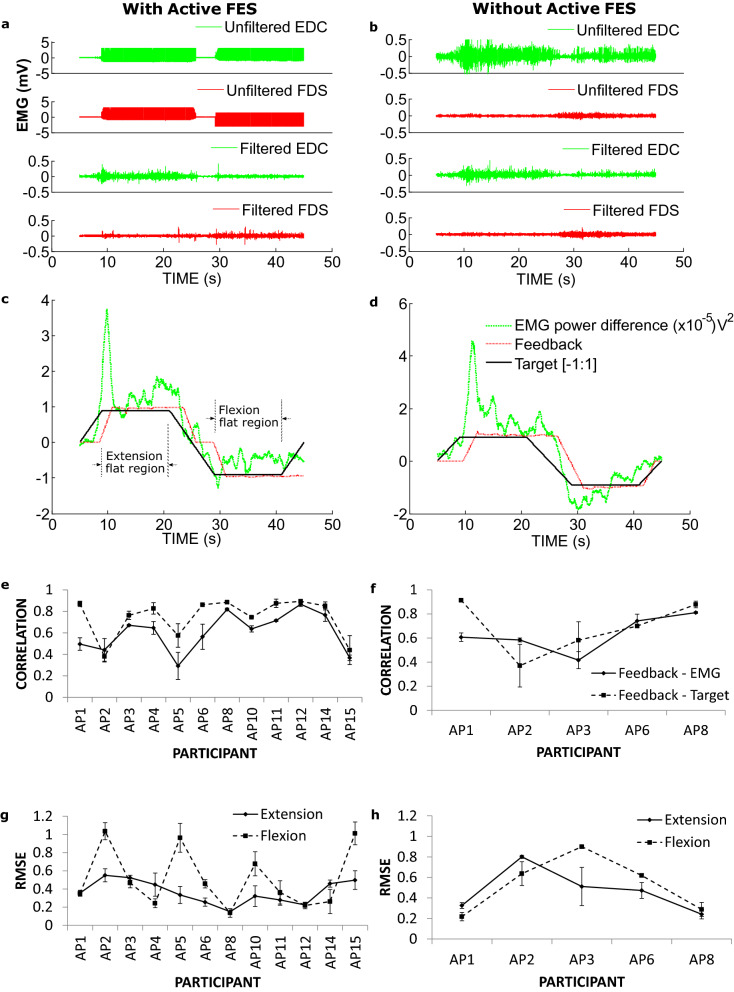
EMG traces and the relationship between EMG power, target and feedback during EMG shape tracing with and without the Active FES system. An example EMG traces recorded when a patient was performing the tracing task (**a**), with the Active FES system; (**b**) without the Active FES system. Feedback traces (computed from the filtered EMGs from (**a**) and (**b**) when a patient was performing the tracing task (**c**), with the Active FES system and (**d**), without the Active FES system. Correlation between Feedback and EMG, and Feedback and Target during the tracing task with (**e**), and without (**f**), the Active FES system. Respectively, (**g**) and (**h**) are the root mean squared error (RMSE) between Feedback and Target within the flat region of the target with and without the Active FES system. The Feedback was computed by subtracting Flexor digitorum superficialis (FDS) pulsewidth from Extensor digitorum communis (EDC) pulsewidth, (EDC_pw_–FDS_pw_). The EMG power difference was computed by subtracting EMG power for FDS from that of EDC (EDC_power_–FDS_power_).

In the first tracing task, participants were told to use hand and wrist movement to follow a* target* shape profile (see Fig. 5c,d) displayed on a computer screen. This task allowed the participants, guided by the feedback, to proportionally vary the EMG from the extensor and flexor muscles during voluntary movement in order to follow the displayed shape. It helps to determine the controllability of the system.

An example of recorded raw (unfiltered) and extracted (filtered) EMG signal is shown for a patient performing the tracing task in Fig. 5a with the Active FES system and in Fig. 5b without the Active FES. How well the patients followed the target was assessed using correlation coefficients between the provided Feedback (visual feedback is a normalised derivative of an applied FES pulsewidth, see the Methods for more details) and extracted EMG (Fig. 5a,b), and also the shape the participants were tracing (Fig. 5c,d). The average correlation coefficient across the participants when using the Active FES to perform the task was 0.61 ± 0.051 between the Feedback and EMG, and 0.75 ± 0.052 between the Feedback and Target (Fig. 5e). Similarly, the average correlation coefficient when the Active FES was not used to perform the task was 0.63 ± 0.068 between Feedback and EMG, 0.69 ± 0.10 between Feedback and Target (Fig. 5f). There was no statistical difference in the correlation coefficients with (n = 12) and without (n = 5) the Active FES (t-test, unequal variance; Feedback ~ EMG, *p* = 0.78; Feedback ~ Target, *p* = 0.62). These results demonstrate that the Active FES was able to extract residual vEMG despite an ongoing FES, that was similar and can be used for a similar control purpose as EMG normally recorded without FES.

Controllability of the Active FES was estimated using the Feedback error with reference to the tracing Target shape within the ***flat region*** (indicated in Fig. 5c). With the Active FES this error, average root mean square error (RMSE) across the participants was 0.37 ± 0.037 (arbitrary unit) during hand and wrist extension and 0.52 ± 0.094 during flexion (Fig. 5g). Similarly, without the Active FES the average RMSE were 0.47 ± 0.096 during extension and 0.53 ± 0.12 during flexion (Fig. 5h). Again there was no statistical difference between task performances with and without the Active FES for extension (*p* = 0.36) and flexion (*p* = 0.92). This supports the earlier results that the Active FES can extract useable vEMG during the FES application. Modulation of EMG output of a pair of muscles in the presence of coactivity to precisely follow a given target may be a difficult task for patients with impaired functions. This and the likelihood of a reduced reaction time for the patients (e.g. cf. EMG power difference traces vs. the target shape in Fig. 5c,d) when responding to changes in the target shape may lead to large RMSE values. Therefore what constitutes a large error here needs to be relative to the RMSE for the version of the task without the Active FES system. Despite the variation in RMSE values especially in the case of flexion with Active FES (Fig. 5g), the limited available data suggest that there is no performance difference compared to the case without the Active FES system.

**Figure 6:**
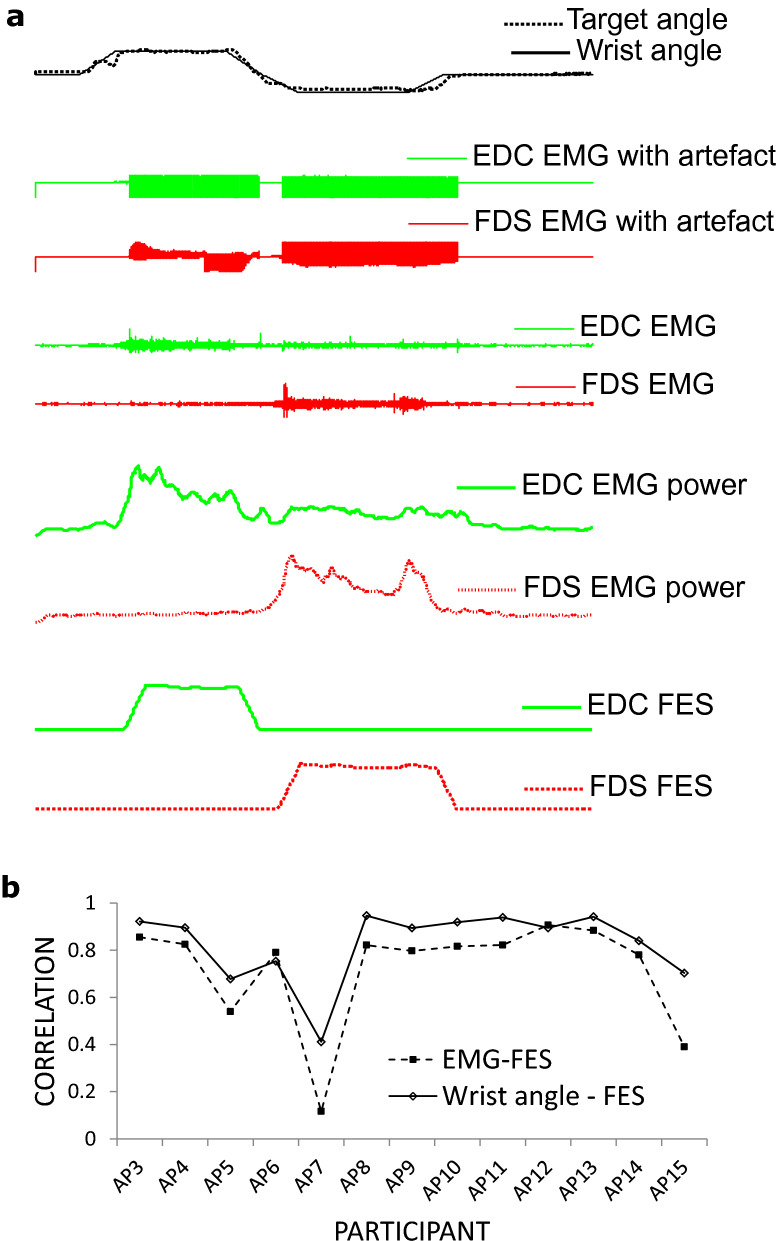
Relationship between EMG, FES and wrist angle during a tracing task. (**a**) Example from a patient on how FES pulsewidth is related to EMG power and consequently the position of the wrist. (**b**) Correlation coefficient between applied FES pulsewidth and EMG power, and wrist angle for individual subjects.

In the second tracing task, the participants were asked to track a target profile using hand and wrist movement but this time were provided with their wrist angle as feedback and allowed to exceed the target height within the flat region. This task enabled the participants to use the feedback to follow the target shape by varying the wrist angle. It helps to determine if the FES intensity is related to intentional movements and secondarily to test for assistive benefit. Figure 6a) shows an example from one participant performing the task. The applied FES pulsewidth was highly correlated with the extracted EMG and consequently the wrist angle (Fig. 6b), with an average correlation coefficient across the participants of 0.72 ± 0.064 for FES pulsewidth vs EMG and 0.83 ± 0.043 for FES pulsewidth vs wrist angle. The result that FES intensity correlates with voluntarily generated movement signals means that the Active FES may be used as a proportional EMG-FES system. The result lends the Active FES to application in movement neurorehabilitation where timely association between intentional movement and related sensory input could lead to better outcome^4^.

**Figure 7:**
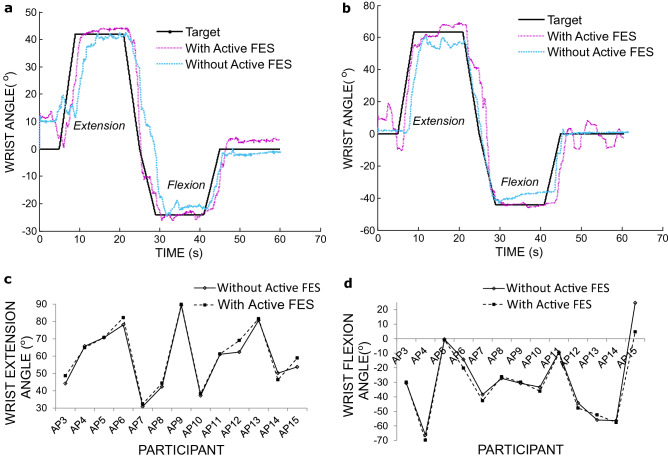
Results of a shape tracing task. (**a**) Example from participant AP8. (**b**) example from participant AP12. (**c**) Maximum wrist angle during extension. (**d**) maximum wrist angle during wrist flexion, with and without Active FES.

Additionally, although it was not the main focus, some patients were able to reach higher wrist angles both for extension and flexion when using the Active FES system for the second task (Fig. 7a,b). In general, for the extension the mean maximum wrist angle was 59.0 ± 4.9°, without and 60.7 ± 5.0°, with the Active FES system (Fig. 7c). For the flexion it was − 29.4 ± 6.9°, without and − 32.2 ± 6.1° with the Active FES (Fig. 7d). There was no statistical difference (2-tailed paired ttest, n = 13) between the achieved wrist angles with and without the Active FES for extension (*p* = 0.051) and flexion (*p* = 0.105). Although these results for wrist angles are not statistically significant, it may be possible for some participants, for the Active FES system to serve as an assistive device as well as a rehabilitation system following further development.

The mean rating by the participants on the ‘Device’ subscale of the QUEST^31^ for the Active FES system was 4.0 ± 0.7 (mean ± SD, max = 5). This score is equivalent to ‘quite satisfied’ on the QUEST scale. The participants most frequently selected *Ease to use, Comfort* and *safety* as the most important items for them. These results demonstrate the capability of the Active FES to work in patients who have minimal EMG.

## Discussions

This study presents a filtering technique to eliminate stimulation and muscle response artefacts during FES in real-time without the need for a special hardware. The presented technique outperformed the comb filtering method effectively eliminating the artefacts to extract a signal which was similar to an original input EMG. The technique was used to implement the Active FES system which was piloted among potential users who have a chronic or acute tetraplegia. Verified using tracing tasks, the Active FES delivered graded electrical stimulation to the participants’ hand muscles in accordance with their intended hand and wrist movements. Since this manner of electrical stimulation may promote active engagement and corticospinal changes, the result suggests that the system could be used for neurorehabilitation of functional movement following neurological disorders.

Devices that can both record EMG and apply FES on the same muscle reported in the literature^27–29^ extract a signal with dead zones due to the use of a blanking window, and reduced bandwidth. They were not designed to completely eliminate the m-wave and post-stimulus decay artefacts and therefore were not able to extract pure vEMG.

We wanted an entirely software-based solution to the problem and therefore investigated an adaptive filtering technique^16,17^ which has been previously studied using a blanking window that creates dead zones in the output. We replaced the hardware pre-processor that provides the blanking functionality with a comb filter^32^ with order that was equivalent to FES frequency. On further investigation in which we studied different variants of the adaptive filter by varying its characteristics including filter order and frame length, we discovered that the adaptive filtering technique can extract vEMG without the need for the pre-processing using the comb filter to eliminate the transient stimulation artefact. We also noted that, in addition to a superior performance over the comb filter, visually the adaptive filter resulted in a signal with a better frequency spectrum. Without the need for a special hardware, blanking window and a high cutoff frequency filter, the present solution allows full bandwidth EMG to be acquired. The solution should also avoid dead zones if the signal is acquired with an amplifier with a suitable dynamic range. Given that no special hardware is required the technique can easily be implemented and extended to any number of channels. The presented technique therefore provides a satisfactory result, a sufficient and reliable solution to the artefact problem with proportional EMG-controlled FES where recording and stimulation is carried out on the same muscle.

We developed the Active FES system and in order to test it we selected typical parameters often used in FES application, including frequency, 25 Hz; current, 10–28 mA and allowed the pulsewidth to vary according to detected movement intention. Although the Active FES included double threshold method^16^ we primarily used and presented the results with the normal single threshold method. The pilot results using these parameters and an order of six for the adaptive filters for tracing tasks showed that the operation of the FES highly correlated with voluntarily generated signals. This shows that the FES operated in accordance with the patients’ hand movement intention. Since the FES is deactivated with absence of movement, the Active FES may motivate active engagement of users. Essentially, the Active FES implements the hypothetically effective method of FES application through peripheral electrical stimulation contiguous and contingent to voluntary motor activity which facilitates neuroplasticity leading to movement rehabilitation^4,33^. Importantly the results also indicated through the QUEST score that the participants accepted the system.

The previously published methods required a special hardware for blanking window functionality, extract noisy or EMG with incomplete frequency information through the use of high cutoff filters^16,17,22–25,27–30^. The required hardware for blanking functionality which may not be readily available, deletes signal samples from the extracted EMG. The number of deleted samples varies according the blanking window length which requires adjustments for optimal performance since the target artefact is non-stationary. The present study shows that the Active FES system provides a method of extracting full bandwidth voluntary EMG during FES. Unlike in previous studies we used simulations to demonstrate that the online extracted EMG is coherent with a noise free version of the signal. A strength of the Active FES system is that no special hardware is required in order to extract the voluntary EMG meaning that it may be easily implemented compared to previous applications of the presented adaptive filtering method^16,17^. The software signal extraction approach does not require a high cutoff high-pass filter and a blanking window which may introduce dead zones as in previous studies. We also showed that the method is effective in a two-channel setup and developed a control system that used the extracted signal from two muscles to augment hand movements by delivering electrical stimulation to the appropriate muscle despite coactivity. In addition to the spectral characteristics of the extracted signal, we presented a method of determining the mean frequency response of the adaptive filter, studied and presented its frequency characteristics. We tested the Active FES among fifteen patients with impaired muscle function in a typical use case where the patients controlled the Active FES system by attempting voluntary movements. Overall this study demonstrates how easily Active FES can be used to extract vEMG for feedback and control purposes representing a significant development compared with past studies. In order to facilitate research and to provide means of implementation and reproduction of our results, the code developed in this study is made available through GitHub (https://github.com/BethelOsuagwu/active-fes).

Brain computer interface systems such as those built with electroencephalogram (EEG) are used for the control of FES both for assistive and rehabilitation purposes^15^. As rehabilitation systems they are able to motivate active engagement during FES therapy which drives motor learning through simultaneous activation of the motor and sensory systems^4–7^. When a user has residual muscle activity, EMG can be used to build similar assistive and rehabilitative systems and given that EMG is less noisy than EEG it could provide a better control signal for FES. Due to the EMG artefact problem during FES application, implementation of EMG controlled-FES has mainly been limited to a triggering technique which has not conclusively shown additional benefit over conventional, passive FES application^8–12^. The triggering technique is also not adequate for control purposes since it will only be able to activate but not deactivate the FES. The present study shows it is possible to implement the proportional EMG-controlled FES system using only software based filters.

Further work on the Active FES includes reducing the delay introduced by the filters and increasing the number of channels in a computationally efficient manner. Although we tested the Active FES among patients who attempted free hand movement, further tests may be required to determine the performance of the method during activities of daily living. Since the extracted signal is coherent with original EMG, there are possibilities for further research which may include studying the effect of stimulation parameters on muscle activities and assessment of reflexes during FES. Stimulations for evoked potential perhaps delivered randomly and out of phase with FES or any repetitive electrical stimulation may be used to study the effect of the stimulation on muscle responses to sharp stimuli during FES. Additionally, the Active FES system can be used in real-time control models and other investigations such as active motor unit, synergy and coherence analyses^34^ for muscles under electrical stimulation.

## Methods

### Participants

Fifteen participants (mean age, 59 years; range 34–76 years) with SCI took part in this study. Neurological level of injury ranged from C2–C7 and AIS ranged from C–D. One participant (AP4) had AIS of A but had a zone partial preservation. Demographic data of participants is shown in Table S2. The participants were required to have residual EMG activity of the flexor and extensor muscles during hand movement. Exclusion criteria included a known neurological condition or comorbidity such as brain injury, and an inability to understand instructions in spoken or written English. Informed consent was obtained from all participants and the study was conducted in accordance with the protocol approved by Yorkshire and The Humber–Leeds East REC and UK Health Research Authority in line with the Declaration of Helsinki. The trial registration number is ISRCTN28779644 and the registration date is 26/01/2018. The registration details can be found at 10.1186/ISRCTN28779644.

Participants agreed, with a Physiotherapist, on which hand to use for the study. Typically if both hands had residual movement the hand a participant believed had the worst impairment was used. Otherwise the hand with some residual movement was used.

To further assess the capacity of the participants, the self-care subscale of Spinal Cord Independence Measure III (SCIM III)^35^, Toronto Rehabilitation Institute hand function test (TRI-HFT)^36^ and also Modified Ashworth scale (MAS)^37^ were administered. SCIM is a subjective scale that measures meaningful functional changes in people with spinal cord injury. TRI-HFT assesses the capacity to perform everyday tasks using the hand. It has five components including object manipulation, rectangular wooden blocks, instrumented cylinder, instrumented credit card and wooden bar, which measure gross motor function. The participant’s scores for SCIM and TRI-HFT are presented in Table S2 and Table S4 respectively. Modified Ashworth scale was used to assess muscle stiffness during passive movement of the upper limb administered by a Physiotherapist. The scale ranges from ‘0 = normal’, ‘1’, ‘1 + ’, ‘2’, ‘3’, and ‘4 = worst’. The participant’s scores are presented in Table S3.

### Setting

The study took place at the National Spinal Injuries Centre (NSIC), Stoke Mandeville Hospital, Buckinghamshire Healthcare NHS Trust, Aylesbury, HP21 8AL.

### Apparatus

All EMG recordings in this study were performed with SX230-1000 EMG sensor using the DataLink data acquisition system (DLK900) by Biometrics Ltd. The DataLink amplifier was set at a sample rate of 1000 Hz. The position of the wrist was recorded using the SG65 Goniometer by Biometrics Ltd. Electrical Stimulation was applied using the Hasomed Rehastim v1 FES system. The stimulation frequency was fixed at 25 Hz, the current was individually set and was typically between 10–28 mA and the pulsewidth was proportional to EMG power. The Rehatrode FES electrodes (sizes 4 × 6.4 cm oval and 5 × 9 cm rectangular) were used to apply the stimulation.

Implementation, simulations, data recording and processing was performed in MATLAB and Simulink (R2014a) using custom code which is available through GitHub.

### Artefacts, filters and Active FES system

#### Recording sample data and noise characteristics

Participants were setup with the Active FES system (see below) where an FES electrodes pair was placed over the Extensor digitorum communis (EDC) and another over the Flexor digitorum superficialis (FDS) muscle. EMG electrodes were placed between each pair of FES electrode on the EDC and FDS muscles. The participants were asked to close and open the hand, and flex and extend the wrist as much as they could while EMG was recorded from the EDC and FDS muscles. The wrist angle was also recorded.

#### Filter design

##### Comb filter

A general feedforward comb filter is given by,

1 $${x}_{c}\left(n\right)=x\left(n\right)+bx(n-L),$$

2 $$L=Fs/f,$$

where here ${x}_{c}\left(n\right)$ is an input EMG including the transient stimulation artefact, post-stimulus voltage decay and m-wave. If $L$ is chosen such that its value is the ratio of EMG recording sample rate to FES stimulation frequency, then the transfer function zeros of the comb filter will align in the frequency axis with the transient stimulation artefact. To cancel out the aligned transient artefact, the comb filter is set to use $b=-1$.

##### Adaptive filter

Assuming that the vEMG can be modelled by a band limited Gaussian noise^17,38^ during usual functional movement, we followed the adaptive filtering technique presented by Sennels and colleagues^17^. Given that the filter is adaptive, we further assumed that it will be capable of removing not only the m-wave as suggested by the authors but also the post-stimulus voltage decay by adapting to the average of overall approximately temporally fixed artefacts. The filter would typical filter out an average of stationary signal components independent of the sources and number of sources. The filter is given by,

3 $$y\left(n\right)={x}_{c}\left(n\right)- \sum_{i=1}^{M}{b}_{i}{x}_{c}(n-iL)$$

where $y(n)$ is the output clean EMG, ${x}_{c}(n)$ is the comb filtered EMG, $L$ is given by Eq. (2 ) for the filter to function correctly. However, unlike in Eq. (1), $b\equiv \widehat{b}\to {b}_{i}$ is a vector with $i=1, 2, 3, \dots M$, where $M$ is the number of previous recording frames that should be considered for computation of the filter coefficients $b$ for the current frame. This filter therefore works by predicting the coefficients $\widehat{b}$ required to estimate the stationary signal components using $M$ previous frames each of length $L$ samples in addition to the present frame (i.e. $M+1$ frames). In accordance with Eq. (3), the predicted coefficients are used to predict the artefact in the previous $M$ frames with the result subtracted from the raw current frame leaving behind vEMG for the current frame.

In order to use this filter correctly we experimented with various values and decided to set *f* = 25 Hz, $M=6$ and used the EMG amplifier at a sample rate of *Fs* = 1000 Hz. We also experimented with setting $L$ to 40 and 80.

#### Simulink implementation of filters

A real-time model was used to implement the two filters in Simulink. The comb filter was implemented using a MATLAB Function block in Simulink by writing the filter’s difference equation in MATLAB code.

For the adaptive filter, signals were buffered in frames of length $L$. When a frame was recorded, the filter equation was solved using LU factorization method implemented in Simulink block from DSP System Toolbox. The model containing the MATLAB scripts, signal routing and Simulink blocks used for the implementation can be found in GitHub repository at https://github.com/BethelOsuagwu/active-fes.

#### Simulation to study the characteristics of the adaptive filter

In order to determine the characteristics and the effective delay due to the adaptive filter, a real data sample of EMG recorded from a patient with SCI was used. The real EMG data used was a sample of EDC EMG data recorded from participant P13 at a sample rate of 1000 Hz. The sample data was duplicated to create two-channel signal with similar characteristics. The two channels were contaminated with simulated stimulation artefact and simulated m-wave, before being passed through the two filters in series combination starting with the Comb filter, and also through only the individual filters using the Simulink implementation.

To simulate the m-wave at 25 Hz, the model given by Sennels and colleagues^17^ was used where α = 200 ± 20 and τ = 20 ± 5 were used. The α and τ were varied within the given range using a uniform random number generator. The stimulation artefact was simulated by introducing a single pulse of length one sample to the real EMG data at 25 Hz. Note that a limitation of this simulation is the lack of inclusion of the post-stimulus voltage decay. The magnitudes of both the simulated m-wave and stimulation artefact were approximately 100 times higher than that of the real EMG in order to represent a worst case noise condition^17^.

The filter length used for the first duplicate channel was the **short filter** length which had $L = 40$ while a **long filter length** which had $L = 80$ was used for a second channel. The filter coefficients used to filter each frame of the input data were stored for 35 s to study the characteristics of the filters. An average of the stored filter coefficients was computed and then used to analyse the frequency and phase responses, and group delay of the filter.

The group delay for the short filter (Fig. 2bc) has a maximum of 9 samples within the approximately linear part of the passband and 73 samples near the stopband. The long filter on the other hand (Fig. 2bf) has a maximum of 45 samples within the passband and 167 samples near the stopband.

Since the adaptive filter in this study was applied after recording a complete frame, to enable the computation of its coefficients, it requires an additional delay equalling the number of samples in a frame. For the short filter this is 40 samples while for the long filter it is 80 samples. Therefore working with the maximum possible delay, the effective delay of the filter is 73 + 40 = 113 samples for the short filter and 167 + 80 = 247 samples for the long filter, which respectively corresponds to 113 ms and 247 ms given our sample rate of 1000 Hz. Similarly the minimum delays are 49 ms for the short filter and 125 ms for the long filter. The maximum delays occur near the bandstops of the filters and therefore apply mainly to the removed signal components (see Fig. 2bc,bf). Therefore the true average delays due to the filters are likely closer to the minimum delays.

#### Filter performance evaluation

Muscles response index (MRI^17^), power reductions and average coherence^39^ were used to assess the filtering performance.

The MRI index measured in dB has a value of zero for a perfect filtering and less than zero for imperfect filtering. MRI_x_ represents the MRI of the input signal while MRI_y_ is for the output signal.

The addition of artefact on vEMG results in a signal with a higher overall power density. An effective filter can reverse the power increase by eliminating the artefact. Therefore we assessed the performance of the filters using their capacity to reduce the power of the input signal. Power reduction was computed first, as a ratio of the powers of the extracted signal and the original clean EMG where a resultant value of 0 dB indicates a perfect filtering. Second, power reduction was computed as a ratio of the powers of the extracted signal and the input EMG with artefacts where a resultant value of 0 dB or higher indicates a poor quality filtering.

Coherence provides a method of assessment of the correlation of the spectral components of two signals where a value of unity indicates a perfect correlation and a zero indicates an absence of correlation. It was used to assess the similarity of the spectral components of the signal extracted by the filters to that of the original input.

#### Active FES system implementation

A real-time Simulink model was developed to implement the Active FES system with two channels. The first channel was for the EDC muscle and the second was for the FDS muscle of the upper limb in the current implementation. To setup the Active FES system on a user, two FES electrode pairs were placed over the EDC and FDS muscles of the upper limb (one on each muscle). EMG electrodes are them placed between each pair of FES electrode on the EDC and FDS muscles.

The power of the filter-extracted EMG was computed and smoothed before being passed to a block that converted it to a reference signal relative to a calibration EMG recorded during maximum voluntary contraction. This block also estimated the direction and type of the intentional movement based on the relative EMG powers on both channels. The reference signal is then passed to another block that converted it to an FES pulsewidth referred to as the computed pulsewidth. The same block also smoothed the computed puslewidth to obtain applied pulsewidth using the filter,

4 $${p}_{a}[n] = {p}_{a}[n-1] + sign( {p}_{c}[n] - {p}_{a}[n-1] ) \times \eta$$

where ${p}_{c}[n]$ is the computed pulsewidth, ${p}_{a}[n]$ is the applied pulsewidth and $\eta$ is a step size. This filter was operated at the same rate as EMG recording i.e. $Fs=1000$ with $\eta =0.1$. The filter ensured smooth rise and fall of the pulsewidth to reduce discomfort on users. The applied pulsewidth was then sent to the stimulator module along with other stimulation parameters to deliver the stimulation. The schematic diagram of this implementation is shown in Fig. 4 and the source code for the project is available from GitHub @ https://github.com/BethelOsuagwu/active-fes.

The developed Active FES system model provides several options that can be used to configure and customise its operations. For example it is possible to set the system to prevent simultaneous activation of the two channels and to dynamically adjust the movement detection thresholds based on the stimulated muscle. A list of the main available configuration options is shown in Supplementary Table S1. A custom graphical user interface with controls to change Active FES parameters including FES settings in real-time was developed to make it easy to setup and alter some of the configuration options during a session.

##### Implementation summary

In the Active FES model, the user begins a movement attempt of the hand and wrist and the resultant vEMG is detected and used to proportionally modulate FES pulsewidth to augment the intended movement such that the intensity of the FES is increased with the EMG power. Artefacts from the FES contaminate the EMG resulting in a noisy signal unsuitable for the control of the FES. But as the entire recorded EMG is passed through the filters the vEMG embedded in the noisy signal is extracted, and used to continue to proportionally control the FES intensity. The model also integrates a biofeedback which continuously presents visual feedback to the user showing an estimation of the voluntary effort from the EMG and the applied FES pulsewidth for the individual channels. An example plot of the input and output of the Active FES model during a typical use is shown in Supplementary Fig. S2.

### Pilot study of the Active FES system in spinal cord injury

#### Design

This was a feasibility study in which each participant was invited to try the Active FES system. The participants attended a maximum of three study sessions with each session lasting for a maximum of 2 h. They were first assessed using various assessment tools during the first session. Depending on their level of impairment which affected the speed of the assessments, participants tested the Active FES system and the study tasks during the first or the second session. Some participants (three) had to attend the third session due to problems with recordings in the previous sessions.

#### Tasks

With and without using the Active FES system, participants were asked to perform two tracing tasks. The tracing tasks were used to demonstrate that the Active FES system can, (1) extract vEMG from a stimulated muscle and (2) use the extracted signal to proportionally control the FES intensity. When the Active FES system was used electrical stimulation from the device was delivered to the flexor and extensor muscles in proportion to their activation level. During the task wrist angle in addition to EDC and FDS muscle EMG were recorded.

In the first task they were ask to trace a target shape displayed on a computer screen. The target shape is shown in figure (Fig. 5c,d). They were instructed to follow the target using hand and wrist movements. They received a visual feedback, in addition to the electrical stimulation, overlaid on the target shape. The feedback was computed by subtracting FDS pulsewidth from EDC pulsewidth, EDC_pw_–FDS_pw_. This task allowed the participants, guided by the feedback, to proportionally vary the EMG from the extensor and flexor muscles during voluntary movement in order to follow the displayed shape.

In the second tracing task, the participants were asked to follow a similar shape as in the first task by opening and closing the hand and extending and flexing the wrist. The height of the shape which the participants were allowed to exceed corresponded to 10% more than their maximum voluntary wrist angles for flexion and extension. Feedback overlaid on the target was in this case provided by plotting the wrist angle on the computer screen. This task enabled the participants to use the feedback to follow the target shape by varying the wrist angle.

#### Procedure

Participants were seated and setup with the Active FES system on the chosen hand. Pairs of FES pads for the Active FES system was placed over the EDC and FDS muscles. On each muscle, one pair of EMG electrodes was placed between the FES pads. A goniometer was placed on the back of the hand to measure wrist angle. Participants were then asked to perform maximum voluntary hand closing and opening, and wrist flexion and extension while calibration EMG and wrist angle were recorded with FES deactivated. The recorded signals were used to calibrate the Active FES system. Participants were asked to then practice with the system before the tracing tasks began. Stimulation parameters were set at a comfortable level for each participant, with the participants involved in deciding an adequate level (see Supplementary Table S1). They tried the tracing tasks in order to familiarise themselves with it. When they felt they were ready the tasks began. There was no limit on the number of trials but the participants attempted the first task on average 3.4 times with the Active FES and 2.3 times without. For the second task, the participants had on average 3.8 attempts with the Active FES and 2.9 attempts without.

Following the tracing task the participants were asked to complete Quebec user evaluation of satisfaction with assistive technology (QUEST, version 2.0)^31^. The QUEST evaluates a user satisfaction on various aspects, of a device including *effectiveness* and *ease of use*, each on a scale ranging from 1 (not satisfied at all) to 5 (very satisfied).

#### Analysis

Data were not available for some conditions and participants because some patients were tired due to their health state during the sessions and they had limited time to take part in studies. The second tracing task was added to the protocol after the second participant. Analyses were performed on available data to determine tracing performances with and without the Active FES system mainly to find out if participants could use the device in the presence of FES. The controllability and performance of the system were assessed using RMSE within the flat region of the tracing target shape, and correlation coefficient between user feedback and each of target shape and power of extracted EMG. Also wrist angle was compared with and without the Active FES system.

### Statistics

The values of coherence between two signals were considered to be significant if they lie above the 95% confidence limit^39^. Student t-test was used to compare the task performances of the participants with and without the Active FES system. Statistical significance level was set at 0.05.

## Supplementary information

Supplementary information.

## Data Availability

The data from this study are available from the corresponding author upon reasonable request.
