# Supplementary material for: Active proportional electromyogram controlled functional electrical stimulation system
Source: Sci Rep. 2020 Dec 4;10:21242. doi: 10.1038/s41598-020-77664-0 (PMC7718906; doi:10.1038/s41598-020-77664-0)
Supplement: Supplementary file 1 — Supplementary information. [file 41598_2020_77664_MOESM1_ESM.docx]

Active proportional electromyogram controlled functional electrical stimulation system

Bethel A. C. Osuagwu^1*^, Emily Whicher^1^, Rebecca Shirley^2^

^1^National Spinal Injuries Centre, Stoke Mandeville Hospital, Mandeville Road, HP21 8AL Aylesbury, UK

^2^Buckinghamshire Healthcare Plastics, Stoke Mandeville Hospital, Mandeville Road, HP21 8AL Aylesbury, UK.

[*bethel.osuagwu@gmail.com](mailto:*bethel.osuagwu@gmail.com)

## Supplementary information

| **Configuration** | **Values** | **Value used** | **Description** |
| --- | --- | --- | --- |
| aFES.Fs | Any* | 1000 Hz | EMG recording frequency |
| aFES.stimCurr | Any | 10 – 28 mA | A two element vector of FES currents values in the respective order of channels. |
| aFES.stimFreq | Any* | 25 Hz | FES frequency |
| aFES.pwFactor | 40 – 400 | 40 - 400 | Used by the reference block to scale a reference EMG power to pulsewidth |
| aFES.pwThresh | 0 – 200 | 100 - 200 | The starting pulsewidth once activation is reached |
| aFES.monoStim | 0 – 1 | 1 | Determines the % level of stimulation allowed on a non-main active Channel. E.g During extension and EDC stimulation is active; then the EDC is the main active channel and any FES applied to the FDS is scaled according to *monoStim*; such that if *monoStim=1*, FDS will not be stimulated, if *monoStim=0.5*, FDS will be stimulated by 50% of its activation value and if *monoStim=0*, FDS will be stimulated by 100% of its activation value. This setting is useful for patients who may have struggle with co-activation and coordination and could simulatnousely activate both channels in no particular order. |
| aFES.singleThresh | 0 – 1 | Used | Two element vector, one for each channel, of % activation threshold with respect to maximum voluntary contraction level. |
| aFES.useDoubleThresh | 0\|1 | 0 | Determines whether a double threshold be used |
| aFES.doubleThresh | 0 – 1 | - | Equivalent to *singleThresh* but applies two activation threshold on each channel. One threshold for activation and another for deactivation. |
| aFES.increaseThreshOnFESBy | 0 – 1 | 0 – 0.1 | Determines the percentage amount to increase a threshold on an active channel making it easy to be deactivated. This is useful for patients who may be struggling to relax after a movement In order to turn deactivate stimulation |
| aFES.wristActSuppON | 0\|1 | 0 | Adjust activation level on a particular muscle based on the wrist angle. E.g when the wrist is extended then the extensor muscle is increased in activation if wristActSuppON =1 |
| aFES.mode | 1 – 4 | 1 – 4 | Used for marking data recorded by the system to identify experimental conditions. 1=normal run,3=tracing run 4=wristtracing. |

Table S1: The main Active FES configuration options. Please see the source code in GitHub @ <https://github.com/BethelOsuagwu/active-fes> for a complete list of configurations. *, Need to consider both FES and EMG recording frequency.


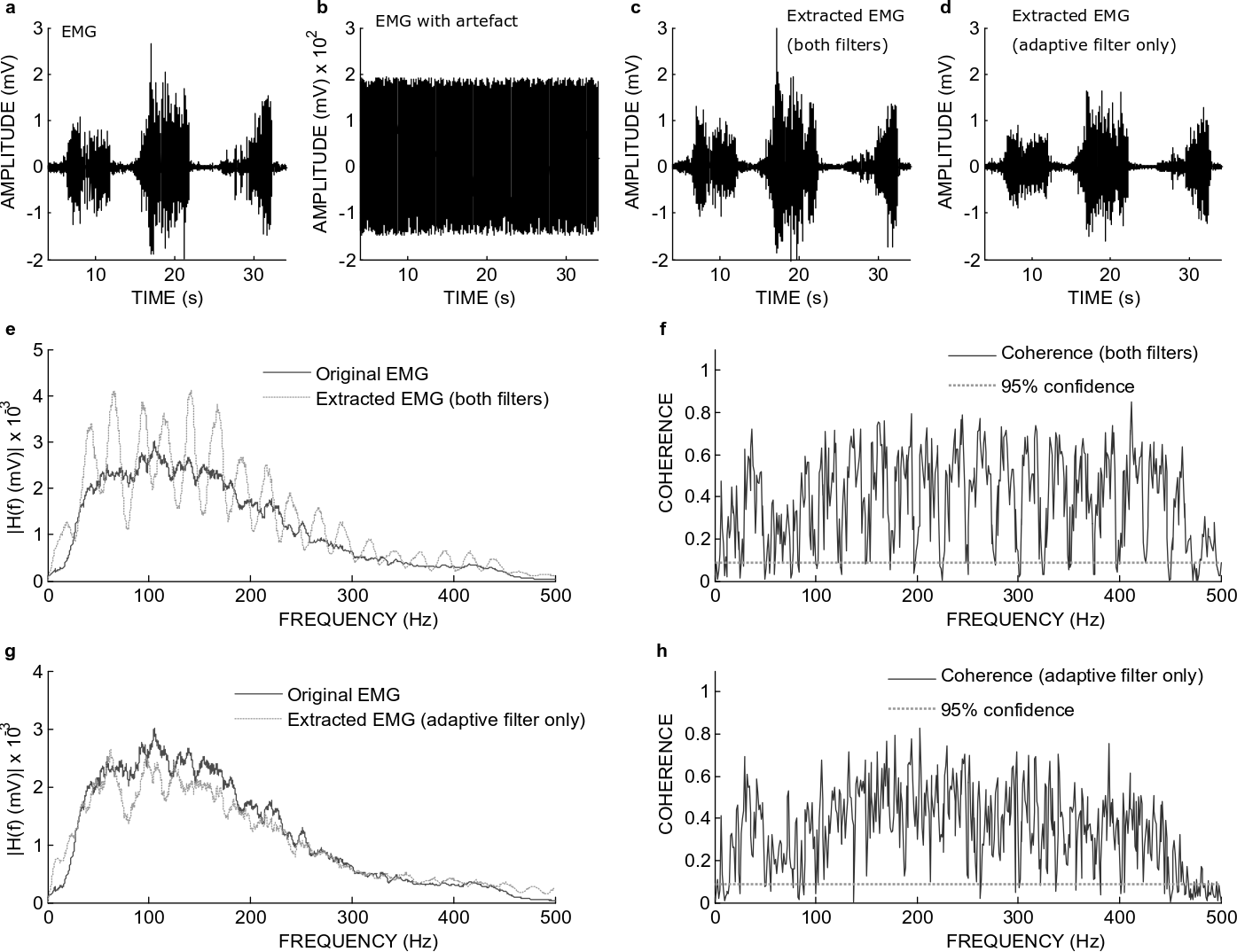


Fig. S1: Filtering performance when a real EMG with added simulated stimulation and m-wave artefacts was filtered with the long adaptive filter, and the comb filter. **a**, Original EMG. **b**, The original EMG with simulated stimulation and m-wave added. **c**, Extracted signal with both the adaptive and comb filter. **d**, Extracted signal with only the adaptive filter. **e**, Spectrum of the original and the signal extracted with both the adaptive and comb filter. **f**, Coherence between the original signal and the signal extracted with both the adaptive and comb filter. **g**, Spectrum of the original and the signal extracted with only the adaptive filter. **h**, Coherence between the original and the signal extracted with only the adaptive filter.


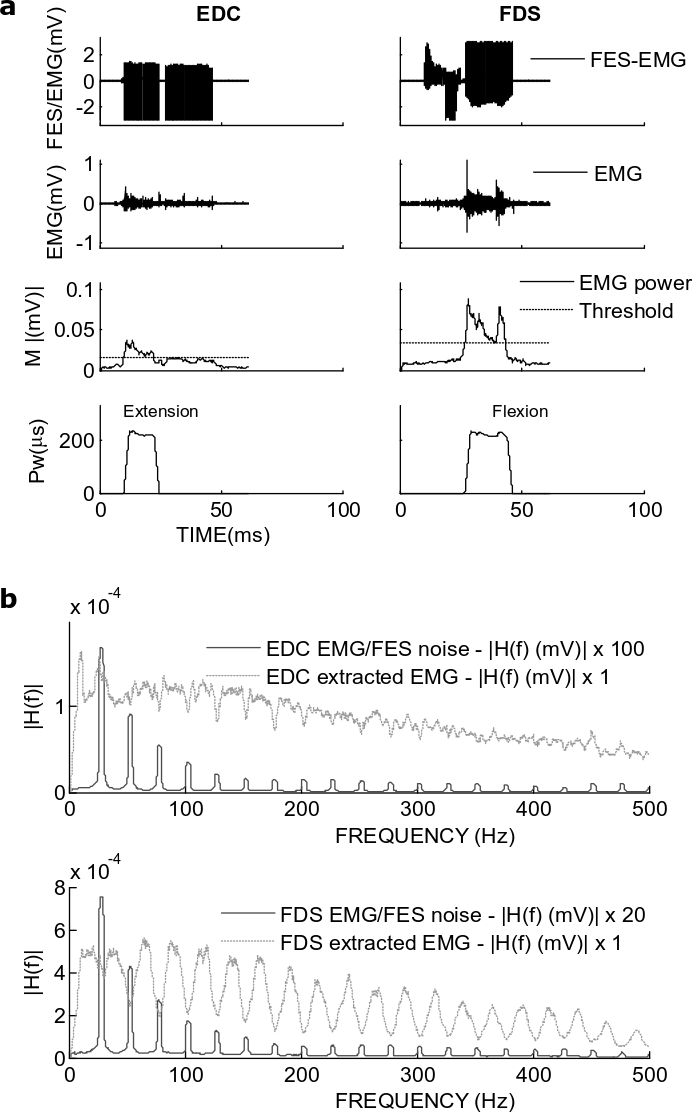


Fig. S2: An example of Active FES model input and output from SCI patients (AP12) performing a tracing task. **a**, The first rows show the input EMG with artefacts and the second rows the EMG extracted from the noisy signal. The third rows show the bandpassed and smooth power computed from the data in second row. The last rows show the FES pulsewidth computed from the EMG power from the third rows. **b**, The spectrum of the noisy and extracted signal from ***a***. EDC, Extensor digitorum communis; FDS, Flexor digitorum superficialis.

| Participant number | Age (years) | Days since injury to initial assessment (Days) | Neurological level of injury | AIS | Study hand | Total SCIM -Self Care |
| --- | --- | --- | --- | --- | --- | --- |
| AP1 | 67 | 183 | C4 | D | R | 8 |
| AP2 | 57 | 124 | C3 | D | R | 10 |
| AP3 | 55 | 315 | C4 | D | L | 12 |
| AP4 | 34 | 382 | C6 | A, Zone partial preservation | R | 7 |
| AP5 | 63 | 158 | C4 | C | L | 3 |
| AP6 | 74 | 446 | C5 | D | L | 12 |
| AP7 | 58 | 60 | C7 | D | L | 8 |
| AP8 | 66 | 159 | C4 | D | R | 5 |
| AP9 (chronic) | 58 | 6834 | C2 | D | L | 7 |
| AP10 | 60 | 140 | C6 | C | L | 3 |
| AP11 (chronic) | 76 | 1485 | C5 | C | L | 4 |
| AP12 | 56 | 66 | C3 | D | L | 18 |
| AP13 (chronic) | 53 | 1537 | C5 | D | R | 17 |
| AP14 (chronic) | 53 | 1836 | C5 | C | R | 7 |
| AP15 (chronic) | 57 | 4687 | C5 | D | L | 9 |

Table S2: Demographics of the spinal cord injury patients. AIS, American Spinal Injury Association Impairment Scale; SCIM, Spinal Cord Independence Measure.

| Participant number | Wrist Flexors | Wrist Extensors | Finger Flexors | Finger Extensors |
| --- | --- | --- | --- | --- |
| AP1 | 1 | 3 | 2 | 2 |
| AP2 | 1 | 1 | 1 | 1 |
| AP3 | 1+ | 1+ | 0 | 0 |
| AP4 | 1 | 1 | 1 | 1 |
| AP5 | 0 | 0 | 1 | 1 |
| AP6 | 1 | 1 | 2 | 2 |
| AP7 | 1 | 1 | 2 | 1 |
| AP8 | 1 | 1 | 1 | 1 |
| AP9 (C) | 0 | 0 | 0 | 0 |
| AP10 | 1+ | 1+ | 2 | 2 |
| AP11 (C) | 0 | 1 | 0 | 0 |
| AP12 | 0 | 1 | 0 | 0 |
| AP13 (C) | 0 | 1 | 0 | 1 |
| AP14 (C) | 0 | 2 | 0 | 3 |
| AP15 (C) | 1 | 2 | 1 | 2 |

Table S3: The Modified Ashworth scale (MAS) for the study participants. MAS ranges from ‘0 = normal’, ‘1’, ‘1+’, ‘2’, ‘3’, and ‘4= worst’. C, Chronic.

| **Participant number** | **1-10. Object Manipulation Component** | **11. Rectangular Wooden Blocks** | 12. Instrumented Cylinder | 13. Instrumented Credit Card | 14. Wooden Bar -> thumb | 14. Wooden Bar -> little finger |
| --- | --- | --- | --- | --- | --- | --- |
| AP1 | 32 | 69 | 5 | 15 | 5 | unable |
| AP2 | 43 | 41 | 5 | 7.5 | 0 | 3 |
| AP3 | 54 | 51 | 17.5 | 15 | 3 | 1 |
| AP4 | 68 | 63 | 7.5 | 47 | 20 | 35 |
| AP5 | 23 | 18 | unable | 1 | unable | unable |
| AP6 | 42 | 27 | 25 | 30 | 15 | 30 |
| AP7 | 68 | 63 | 10 | 17.5 | 1 | 5 |
| AP8 | 58 | 63 | 9 | 23 | 15 | 25 |
| AP9 (OP) | 35 | 36 | 2.5 | 5 | unable | unable |
| AP10 | 62 | 54 | 4 | 24 | 5 | 15 |
| AP11 (OP) | 16 | 11 | unable | 10 | unable | unable |
| AP12 | 70 | 63 | 12 | 27 | 5 | 10 |
| AP13 (OP) | 68 | 63 | 22.5 | 35 | 27 | 30 |
| AP14 (OP) | 65 | 63 | 25 | 37.5 | 2 | 21 |
| AP15 (OP) | 50 | 54 | 12 | 20 | 5 | 10 |

Table S4: Scores of Toronto Rehabilitation Institute Hand Function test (TRI-FHT) for the SCI patients. OP, Outpatient.
